# TDP‐43 pathology in pure LATE‐NC differs from when coexisting with Alzheimer's Disease

**DOI:** 10.1002/alz70855_104302

**Published:** 2025-12-28

**Authors:** Sandra O. Tomé, Klara Gawor, Christine A.F. von Arnim, Rik Vandenberghe, Peter T Nelson, Dietmar Rudolf Thal

**Affiliations:** ^1^ Laboratory of Neuropathology, KU Leuven, Leuven, Vlaams‐Brabant, Belgium; ^2^ University Medical Center Göttingen, Göttingen, Germany; ^3^ UZ Leuven, Leuven, Belgium; ^4^ Laboratory for Cognitive Neurology, KU Leuven, Leuven, Leuven, Belgium; ^5^ University of Kentucky College of Medicine, Sanders‐Brown Center on Aging, Lexington, KY, USA; ^6^ Leuven Brain Institute, Leuven, Belgium

## Abstract

**Background:**

Limbic‐predominant age‐related TDP‐43 encephalopathy (LATE) has been recently recognized as a cause of dementia in the elderly. LATE and Alzheimer's disease (AD) share similar clinical features, and their neuropathological changes—LATE‐NC and ADNC—commonly co‐occur. Still, the morphological and molecular features of TDP‐43 lesions in dementia remain under‐researched.

We investigated TDP‐43 lesion morphology and immunoreactive species in pure LATE‐NC (degree of ADNC: low/not), and ADNC cases with LATE‐NC (ADNC+LATE‐NC, degree of ADNC: moderate/high).

**Method:**

We performed immunohistochemistry in paraffin‐embedded tissue from the hippocampus, amygdala, temporal (perirhinal) and frontal cortices of 70 human autopsy cases including 20 non‐demented controls, 34 demented ADNC+LATE‐NC and 16 demented pure LATE‐NC cases. We semi‐quantitatively assessed the severity of neuronal cytoplasmic inclusions (NCIs) and dystrophic neurites (DNs) using antibodies against several TDP‐43 species: pS409/410, pS409/pS403, pS403/pS404 and C‐ and *N*‐terminal TDP‐43.

**Result:**

Pure LATE‐NC cases usually displayed a band‐like pattern of dystrophic processes in the hippocampus, generally extending from CA1/2 to subiculum. This “LATE”‐like pattern was present in 81% of pure LATE‐NC cases and only 18% of ADNC+LATE‐NC cases. The remaining pure LATE‐NC cases showed a similar pattern, although less severe. Consistently, in multiple linear regressions analyses controlled for age and sex, pure LATE‐NC cases showed more severe burden of DNs in the CA1 (*p* <0,0001, R^2^ = 0,59), amygdala (*p* = 0,0068, R^2^ = 0,52) and temporal cortex (*p* = 0,022, R^2^ = 0,46) compared to ADNC+LATE‐NC, while ADNC+LATE‐NC cases showed higher predominance of NCIs in the amygdala. Moreover, pure LATE‐NC cases had higher burdens of pS403/pS404 and full‐length TDP‐43 species.

**Conclusion:**

The histopathologic features of TDP‐43 proteinopathy differs in pure LATE‐NC vs when coexisting with ADNC, indicating that LATE‐NC tends to be modified in the presence of moderate‐high levels of ADNC. These data suggest that different mechanisms may underlie TDP‐43 aggregation in different amnestic dementias, and that coexisting pathologies can influence each other.

